# Mixture of Digitalis and Iron for Cardiac Weakness, with Dilation of Ventricles

**Published:** 1879-10

**Authors:** 


### EXCERPTA.

MIXTURE OF DIGITALIS AND IRON FOR CARDIAC WEAKNESS, WITH DILATION OF VENTRICLES.

℞ Tr. ferri perchlorid.................... ʒij

Syr. zingiberis.................... ʒvj

Inf. digitalis.................... ℥v

M. Tablespoonful three times daily.

*Oxalate of Cerium for Vomiting of Pregnacy.*

℞ Cerii oxalatis.................... gr. xxiv
Ext. hyoscyami....................gr. xxxvi
M. Et ft. pil. No. xii

Take one twice a day.

*Sleeplessness in Uterine Disorders.*—When patients complain of nervousness or of sleeplessness, the potassic bromide must be given, either alone or in combination with other remedies. A cheap mixture, much thought of by our patients at the University clinic, is the following:

℞ Pulv. ferri sulphat.................gr. xxx

Potassii bromidi..................} aa ℥j

Rad. calumbæ contus.............}

Aquæ bullientis..................... Oj

Steep for twenty-four hours and then strain.

Sig. One tablespoonful in a wineglassful of water just before or after each meal.

I cannot say much for the palatableness of this infusion nor more for its pharmaceutical elegance; but it does good, and we therefore give it largely to our poor patients. The iron and the potash in it may be increased or lessened, or the former may be left out, as the case may be. The zinc valerianate given thrice daily in doses of from two to four grains is one of our best nervines. For a better class of patients the following antispasmodic mixture can be prescribed with very general satisfaction:

℞ Elixir humuli........................fl. ℥j

Elixir ammoniæ valerianat....} aa fl. ℥ ss

M. Syrupi lactucarii.............}

Sig. One dessertspoonful at bed-time, or during the day when needful.

*Ergot and Aloes for Amenorrhœa from Atony of the Uterus.*

℞ Tr. ergot............................ ʒij
Decoct. aloes comp.................*ad* ℥viij
M. Two tablespoonfuls twice a day.—*Hospital Gazette.*

